# Lead exposure in homes as modifying factors of blood lead levels among young children in Bihar, India

**DOI:** 10.1007/s10661-025-14396-6

**Published:** 2025-07-29

**Authors:** Emily Nash, Yi Lu, Stephan Bose-O’Reilly, Ambrish Kumar Chandan, Lavanya Nambiar, Meenakshi Kushwaha, Given Moonga, Gordon Binkhorst, Kumar Bhaskar, Promila Sharma Malik, Sumi Mehta, Ashok Kumar Ghosh, Arun Kumar, Mohammad Ali, Abhinav Srivastava, Gabriel Sanchez Ibarra, Daniel Kass

**Affiliations:** 1 Pure Earth, 475 Riverside Drive, Suite 860, New York, NY 10115 USA; 2 Vital Strategies, 100 Broadway, 4Th Floor, New York, NY 10005 USA; 3 Institute and Clinic for Occupational, Social and Environmental Medicine, University Hospital, LMU Munich, Ziemssenstr. 5, 80336 Munich, Germany; 4 Department of Infectious and Tropical Medicine, University Hospital, LMU Munich, Munich, Germany; 5 Mahavir Cancer Sansthan & Research Centre, Patna, Bihar India

**Keywords:** Lead, Child, Exposure assessment, Blood lead level, India, Home environment

## Abstract

More than 275 million children in India have elevated blood lead levels (*BLL*s). Previous studies in India have focused on children living in highly polluted areas. In addition to industrial sites, children are exposed to lead in their homes. The study aims to identify sources of lead exposure in a sample of children living in Bihar by assessing lead levels in the children’s homes and products and their association with blood lead levels (*BLL*s). The study used a subset of a statewide *BLL* study in Bihar, India. From the larger sample, 150 children were selected, including those with a *BLL* ≥ 20 µg/dL and a random sample of those below this level. Blood samples from children aged 13 to 60 months were analyzed using the LeadCare II analyzer. A home-based assessment (HBA) was conducted to evaluate lead in soil, drinking water, paint, metal and ceramic cookware, spices, cosmetics, and toys. Lead levels were determined using a portable X-ray fluorescence analyzer and laboratory-based analyses. HBA results were compared with local and international limits. Sampling revealed elevated lead levels in metal foodware and spices. After adjustment, the odds of elevated *BLL* were associated with lead content in spices only (*aOR* = 1.35, 95% *CI* 1.17, 1.58). Elevated lead levels in spices and metal foodware are common in Bihar, India. To protect children’s health, measures are needed to reduce lead exposure, including enforcing regulations on lead content in spices, implementing policies, and monitoring metal foodware items, as well as building public awareness.

## Introduction

Exposure to lead poses severe health risks, particularly for children. Lead exposure affects children’s brain development and can result in a reduction in intelligence quotient (IQ) and behavioral impacts such as diminished attention span and increased antisocial behavior, as well as lower educational and lifetime earnings achievement (World Health Organization, 2021). Lead exposure can also lead to anemia, hypertension, renal damage, seizures, and impacts on the immune and reproductive systems (World Health Organization, 2021). It is believed that many of the neurological and behavioral effects of lead are irreversible (Bose-O’Reilly & Landrigan, 2021; World Health Organization, 2021).

Young children are especially vulnerable to lead poisoning due to both behavioral and physiological factors. Behaviors like hand-to-mouth activity, ingestion of non-food items, and crawling increase the likelihood of contact with and ingestion of lead present in dust, contaminated soil, or consumer products (Bellinger, 2004; World Health Organization, 2021). In addition, the proportion of ingested lead that is absorbed is significantly higher in children compared to adults (Bellinger, 2004).

Further contributing sources of exposure vary between and within countries (Bose-O’Reilly & Landrigan, 2021). Therefore, identifying potential contributors to lead exposure, particularly within the home environment where young children spend the majority of their time, is crucial. In India, identified or suspected sources have included elevated lead levels in soil and dust from industrial sites (e.g., lead-acid battery manufacturing, and recycling), lead-based paint, lead-contaminated spices, consumer products, food, and water (Ansari et al., 2020; Borah et al., 2010; Brown et al., 2022; Forsyth et al., 2024; Keosaian et al., 2019; Kumar et al., 2022; Mahdi et al., 2020; Mohanty et al., 2013; Rashid et al., 2019; Vishwanath et al., 2012).

An estimated 275 million children in India have blood lead levels (*BLL*s) exceeding 5 µg/dL (Rees & Fuller, 2020), a globally recognized threshold requiring intervention (World Health Organization, 2021). The associated disease burden is substantial, particularly concerning intellectual disability outcomes in children (Ericson et al., 2018). The loss of an estimated 7 million disability-adjusted life years (DALYs) and 232,500 deaths were attributed to lead exposure in India alone in 2019 (Murray et al., 2020). These health impacts translate to an estimated annual loss of US$236 billion, equivalent to 5% of the country’s gross domestic product (GDP) (Attina & Trasande, 2013).

Bihar, located in eastern India, is the third most populous state in the country. While it has experienced substantial improvements in recent years, Bihar has the highest proportion of its population living below the poverty level of any Indian state (NITI Aayog, 2023). Several previous studies have examined *BLL*s among children in Bihar, specifically in its capital and largest city, Patna. A recent assessment of *BLL*s of school children across 10 cities in India reported that the subjects in Patna had median *BLL*s of 9.7 µg/dL (Kumar et al., 2025). A pilot study of *BLL*s in children close to an informal lead-acid battery recycling workshop in Patna showed a median *BLL* of 19.2 µg/dL for children (Ansari et al., 2020). A larger follow-up of 135 children in Patna examined *BLL*s and exposure sources among children living close to lead-acid battery operations and those living further away (Brown et al., 2022). The difference in *BLL*s between the two groups was not statistically significant, with an overall geometric mean *BLL* of 11.6 µg/dL; this study found that lead levels in house dust and spices were the most likely to increase blood lead levels.

Given the prevalence and severity of lead exposure identified among children in Patna, the current study expands the geographic coverage to seven additional districts within the state of Bihar, capturing both rural and urban areas, to characterize contributing risk factors among a state-representative sample.

## Methods

### Study area and population

Between December 2022 and March 2023, Vital Strategies and Pure Earth assessed *BLL*s among children under the age of five and their pregnant mothers in eight districts in Bihar, India (Lu et al., 2024). Pure Earth is an international non-profit organization dedicated to solving pollution problems in low- and middle-income countries. Vital Strategies is a global health organization partnering with governments and civil society in 73 countries to implement evidence-based solutions for critical public health challenges.

**Fig. 1:**
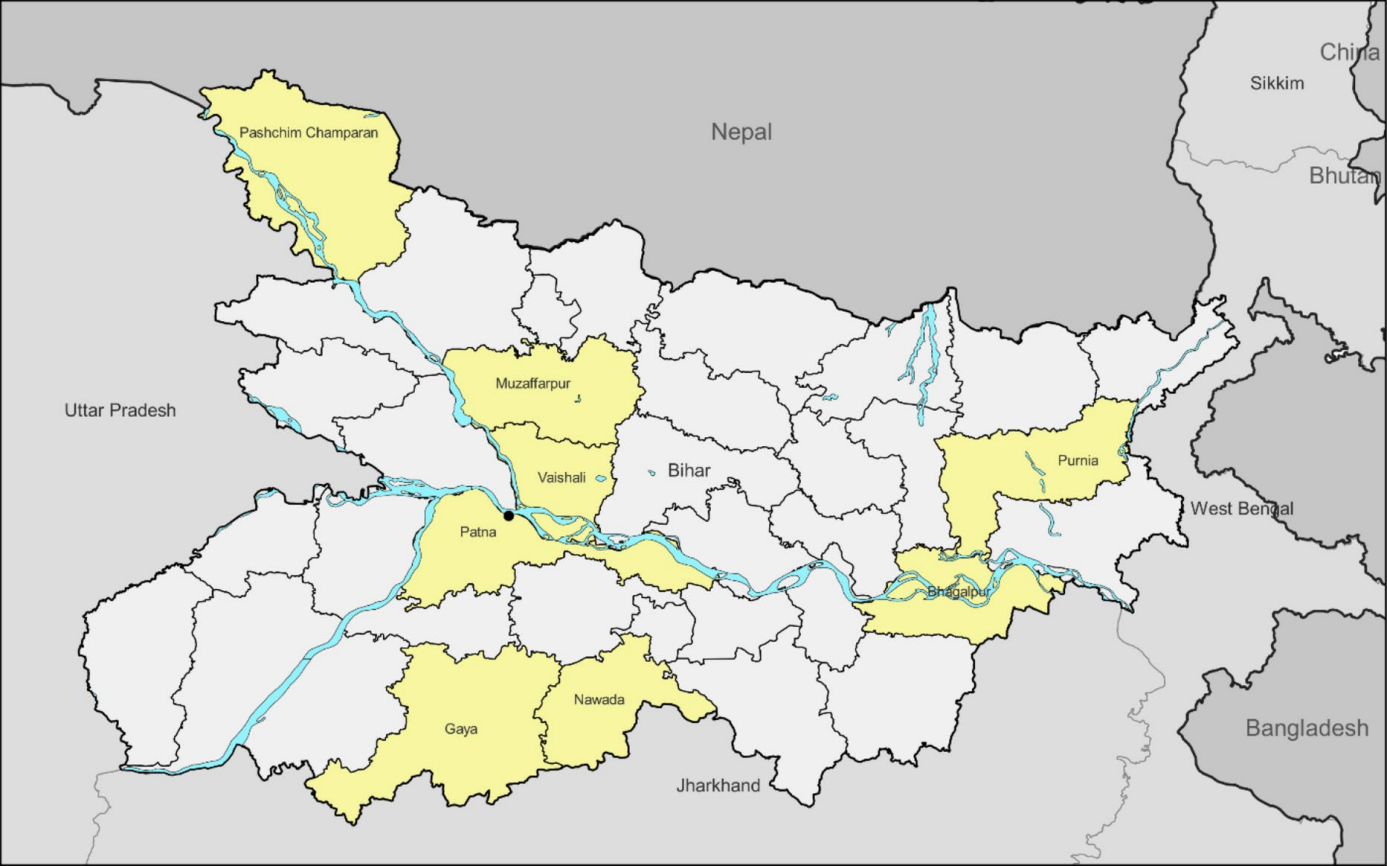
Eight districts in the state of Bihar are included in the current study (highlighted)

The primary investigation was carried out as a cross-sectional study in Bihar to understand the prevalence of elevated *BLL*s among children in Bihar. A multistage sampling design was used for the selection of a state-representative sample of children (*N* = 697) and a convenience sample of pregnant women who were also mothers of enrolled children (*N* = 55). First, 8 districts across Bihar were selected to ensure geographic representativeness (Fig. 1). In the selected districts, 2 to 4 wards or villages were selected using probability proportional to size based on the population reported in the 2011 population survey. Urban areas were intentionally oversampled through the selection of 4 urban wards from districts in the state’s most populous city, Patna. Random walk sampling was used to select households within these wards or villages for *BLL* testing and a questionnaire designed to collect information on factors that may be related to a child’s lead exposure at home (Lu et al., 2024). A subset of participating households was referred for home visits to gather quantitative environmental data on potential sources contributing to elevated *BLL*s.

All households with a participating child with (*BLL*s equal to or greater than 20 µg/dL or with both a participating child and pregnant mother underwent a comprehensive home-based assessment (HBA) to identify potential lead sources within their homes, with informed written consent from the caregiver. Additionally, a 30% random sample of children with *BLL*s below 20 µg/dL was selected to create a comparison group. In total, assessments were conducted at 150 households.

### Health data

Capillary blood was collected from the participating child and analyzed by LeadCare II, a portable analyzer, during a home visit. Information on household characteristics was collected through computer-assisted personal interviews with primary caregivers. More details on the method and results are reported elsewhere (Lu et al., 2024).

### Environmental data

Within the selected households, a standardized approach was applied by a Pure Earth field team to measure lead levels in environmental parameters (soil and water) and consumer products (cookware, spices, paint, ceramics, toys, and cosmetics) in order to evaluate potential sources of lead in the child ‘s home environment (for more details, see Brown et al., 2022). The field crew comprised two teams of six investigators, each with backgrounds in environmental health and social work; these investigators administered the survey, took readings using a portable X-ray fluorescence analyzer (XRF), collected samples, and recorded data.

Where possible, one or more samples were collected for each sample type from a household. Except for drinking water, all aspects of the assessments were carried out using a portable XRF (Thermofisher Niton XL3t 700 s), providing real-time readings in the field. Where multiple readings were taken for any given sample type, the highest reading was used in the analysis. The limit of detection (*LOD*) of the XRF can vary depending on the sample medium being analyzed and selected instrument settings (Thermo Fisher Scientific, 2013). For soil, spices, cosmetics and toys, an *LOD* of 3 ppm was used in this analysis; for metal foodware, 20 ppm; for ceramics, 10 ppm; and for paint, 0.18 µg/cm^2^. In total, 970 samples were assessed via XRF. Lead levels in drinking water were analyzed in a commercial laboratory, using inductively coupled plasma-mass spectrometry (ICP-MS) with a *LOD* of 5 parts per billion (ppb) (Envirochem Research & Test Labs, Lucknow, India).

### Reference levels

To provide context to the lead content found in the various environmental and consumer product samples, a “reference level” for each sample type was selected (Table 1). Existing Indian standards were used where possible. Where such national standards do not exist, standards from the USA were applied. For ceramic and metal foodware, existing standards typically apply to the amount of lead that leaches from the items, as opposed to the lead content in the item itself. Field testing of leachable lead in foodware was not possible. Based on ongoing work of Pure Earth on leaching of lead from foodware, a reference level of 100 ppm for all types of foodware was applied for this assessment. Reference levels for all sample types are listed in Table 1.

**Table 1:** Reference levels for each sample type

Sample type	Reference level	Source
Ceramic foodware	100 ppm	^a^
Cosmetics	20 ppm	Central Drugs Standard Control Organization et al., (2020)
Metal foodware	100 ppm	^a^
Paint surfaces	1000 µg/cm^2 b^	United States Department of Housing and Urban Development/United States Environmental Protection Agency (2012)
Soil	200 ppm	United States Environmental Protection Agency (2024)
Spices	10 ppm	Food Safety and Standards Authority of India et al., (2011)
Toys	100 ppm	United States Consumer Product Safety Commission (2023)

^a^A reference level of 100 ppm was applied for ceramic and metal foodware based on ongoing leachability research, performed by Pure Earth. As of the writing of this article, the Bureau of Indian Standards has started to apply a certification process for aluminum cookware, requiring that it contain less than 500 ppm

^b^India has a standard of 90 ppm for lead content in paint (Ministry of Environment, Forest and Climate Change, Regulation of Lead Contents in Household and Decorative Paints Rules, (Ministry 2016)). For lead that has already been applied to a surface, such as a wall, the XRF best captures results using a mass/area unit, rather than ppm

### Ethical clearance, data management, and data protection

The study was conducted after obtaining approval by the BRANY (Biomedical Research Alliance of New York) Institutional review board located in the US (Protocol Number: 22–176-522) and the ethics committee of the Indian Council of Medical Research — Rajendra Memorial Research Institute of Medical Sciences located in Patna, Bihar (approval letter no. RMRI/EC/54/2022, dated 21/09/2022). The study was carried out in accordance with the Code of Ethics of the Declaration of Helsinki for experiments involving human subjects. Written informed consent was obtained from parents of participating children by researchers who were native Hindi speakers. A copy of the consent form was given to the parents of the participants.

For testing the *BLL* of a child, the parents or legal guardians provided informed consent to complete the interview and carry out blood sampling. A separate form was used for seeking consent for the HBA and an assigned team member explained the purpose of the study to the head of household/guardian including the risks and benefits of the study before seeking consent.

Any data collected on paper was stored in a locked file cabinet in a locked office. The questionnaire data entered and saved in the tablet were exported to the computer and linked with the *BLL* data using a unique ID and saved in a password-protected dataset and kept safeguarded. Participants and households in this linked dataset were identified and referred to by the unique ID only to protect confidentiality. A central list containing unique ID and name/address/contact information was kept separate from the linked dataset. The name, address, and contact information collected during blood testing were provided to Pure Earth to refer the participant for an HBA, if selected. All data sets and reports were stripped of personal identifying information. All field personnel, including local staff, were trained on proper interviewing techniques and on human subject research ethics.

### Variables

Our model uses a dependent binary variable for elevated *BLL* with a threshold of 10 µg/dL. A *BLL* of 20 µg/dL was used to automatically trigger the HBA in order to ensure the most severe cases were included in the sample population and to ensure an adequate comparison group. For the statistical analysis, however, a *BLL* of 10 µg/dL was used; while this is higher than the WHO’s current level for intervention of 5 µg/dL, background levels in Bihar are currently too high to make this WHO recommendation a useful threshold for analysis. Independent variables included in the model are lead levels measured in different environmental samples analyzed in or within the immediate vicinity of each participating child’s home. Among the eight types of environmental samples tested, we selected four types with considerable sample size (more than half of included households), more uniform materials (for example, we excluded readings of furniture), and without a large percentage of samples below detection limits. These four variables are metal foodware, paint on large surfaces, spices, and soil. If multiple items of the same type (e.g., two pieces of metal foodware) were tested in one household, the maximum value is used for the household. Lead content in environmental samples was log transformed using a base of 2 before inclusion in the model due to a skewed distribution and for ease of interpretation.

### Statistical methods

Data were analyzed using Excel, SPSS 27, and R. Both geometric and arithmetic means were reported due to the skewed distribution of lead in blood and the environmental samples, and due to the common use of arithmetic means in producing pooled estimates in systematic reviews. Samples reporting lead levels below the *LOD* were assigned the value of *LOD*/√2. Generalized logistic regression analyses were used to calculate odds ratios (*ORs*) and 95% confidence intervals (*CI*s) for associations between lead in each environmental sample and elevated blood lead levels (*BLL* ≥ 10 µg/dL). The final model for each environmental sample adjusted for important confounders identified from literature including children’s age in months (continuous), sex (male/female), primary caregivers’ education by category (illiterate, school education, and higher education), socioeconomic status (having a below-poverty-line (BPL) certificate or not as a binary variable), and urbanicity (urban/rural).

## Results and discussion

In total, 150 children were assessed for both *BLL*s and lead in their home environment. Characteristics of sampled children and households are presented in Table 2, grouping the children with *BLL*s below and above 10 µg/dL.

**Table 2:** Characteristics of participants by *BLL* (below and greater than or equal to 10 µg/dL)

Characteristics	*BLL* < 10 µg/dL (*n* = 97)	*BLL* ≥ 10 µg/dL (*n* = 53)
Average age in months (range)	38 (25–52)	52 (30–59)
Gender		
Male	46 (47%)	31 (58%)
Female	51 (53%)	22 (42%)
Caregiver’s education		
Illiterate	15 (16%)	7 (13%)
School education	68 (71%)	41 (77%)
Higher education	13 (14%)	5 (9.4%)
Unknown	1	0
Has below-poverty-line certificate	60 (62%)	21 (40%)
Area type		
Rural	82 (85%)	27 (51%)
Urban	15 (15%)	26 (49%)

### Results of the environmental and consumer product samples

For each sample type included in the model, we determined how many households had a reading above the reference level; we also calculated the percentage of households with a reading exceeding the reference level among households with at least one reading in that sample type (Table 3).

**Table 3:** Number of households with environmental samples above reference level and percentages of households exceeding this level among households with at least one reading in that category

Sample type	# Above reference level (%)
Metal foodware	132 (95%)
Spice	69 (54%)
Soil	1 (1%)
Paint on large surfaces	1 (1%)

Cookware made of metal, especially low-cost, locally manufactured items, has been identified as containing lead that may leach into the food during cooking (Brown et al., 2022; Fellows et al., 2022; Weidenhamer et al., 2017, 2023). Predicting the amount of lead leaching from a specific pot is challenging without dedicated testing (Ali Sultan et al., 2023). There are currently no established international standards for the total lead content in metal foodware. In 95% of the examined households, metal foodware was found to contain lead above the reference level of 100 ppm, indicating the potential for lead to leach at a concerning concentration. It should be noted that this category included some items beyond pots or pans, such as plates and utensils, which may not be heated or may have shorter contact time with food, thus potentially posing a lower human health risk.

**Fig. 2:**
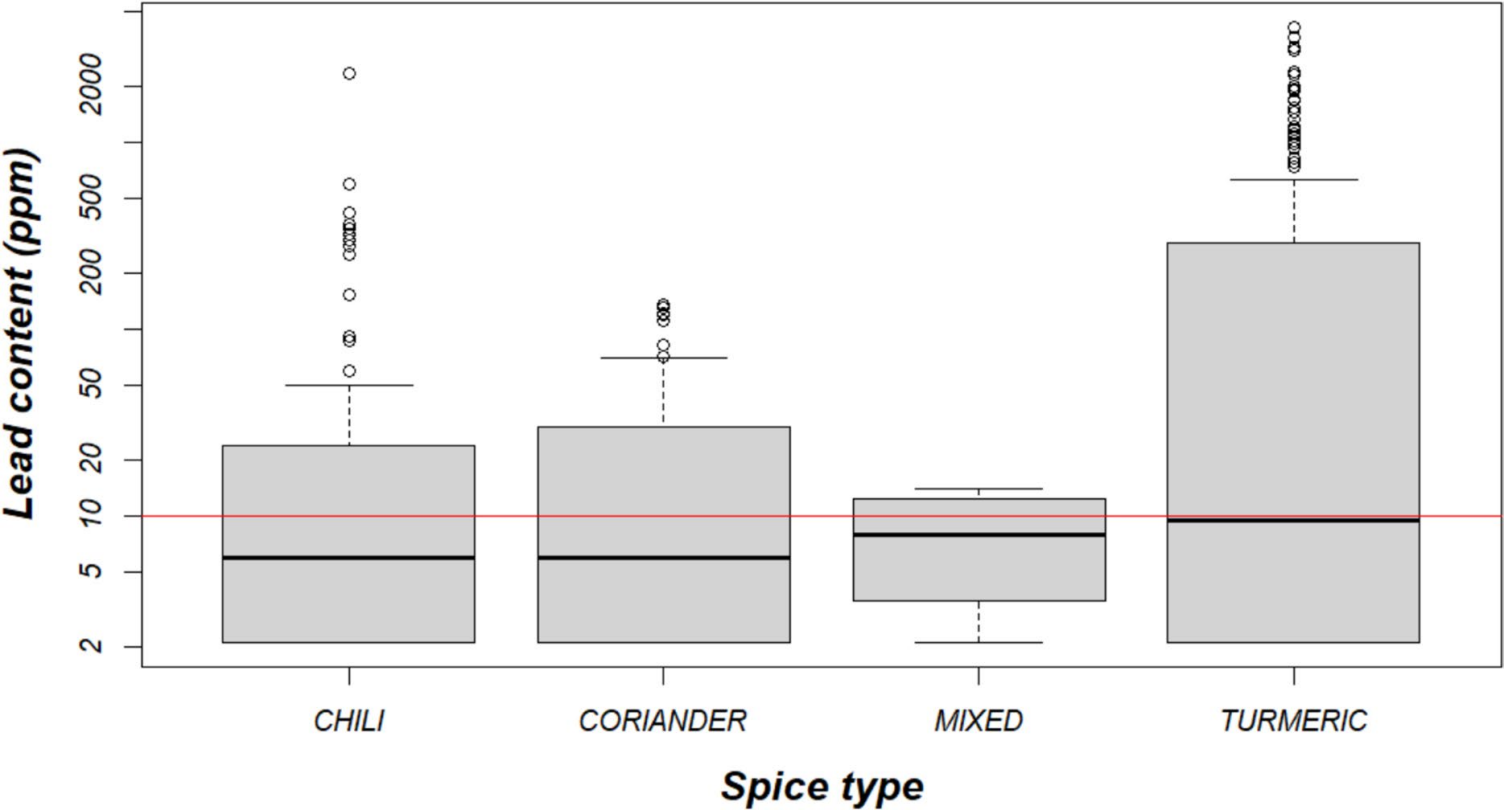
Distribution of lead in spice samples (*n* = 292) by spice type, compared to threshold level of 10 ppm (red line). *Y*-axis is log-scale

Spices in South Asia have been discovered to be adulterated with lead chromate to enhance their color (Baig et al., 2019; Brown et al., 2022; Forsyth et al., 2023, 2024; Gleason et al., 2014; Nordin & Selamat, 2013; Senanayake et al., 2013). This issue was previously identified in Patna, Bihar, but had not yet been confirmed in other parts of the state (Brown et al., 2022; Forsyth et al., 2024). In the current study, spices with lead content exceeding the India Food Safety Authority’s established limit of 10 ppm were identified in all 8 districts, with a prevalence ranging from 8% of spice samples to as high as 76% (Food Safety and Standards Authority of India, 2011). Furthermore, elevated lead levels were detected in all four spice types assessed—chili, coriander, turmeric, or mixed spices (Fig. 2). Turmeric exhibited the highest lead levels among the different spices types, with a median of 9 ppm and a maximum of 4139 ppm, surpassing the regulatory standard by more than 400 times. In 54% of the households surveyed, at least one spice was found to exceed the reference level of 10 ppm.

Readings of soil were obtained from an area directly outside the residence (1 to 4 readings). Out of 79 households tested, only one had soil levels above 200 ppm, the recently revised United States Environmental Protection Agency’s screening level for residential soil (United States Environmental Protection Agency, 2024). This sample had an exceptionally high value of 1990 ppm. The median soil lead level of households of children with elevated *BLL*s is 22 ppm, while the median level of households of children with lower *BLL*s is 17 ppm, indicative of background levels (Table 4).

**Table 4:** Lead levels in household environmental samples by *BLL* among children (*N* = number, *LOD* = limit of detection (20 ppm for metal foodware, 3 ppm for spice and soil, and 0.18 µg/cm^2^ for paint), *GM* = geometric mean, *AM* = arithmetic mean, and *SD* = standard deviation)

Environmental sample	% Below *LOD*	*BLL* < 10 µg/dL					*BLL* ≥ 10 µg/dL				
		*N*	Median (min and max)	*GM* (*SD*)	*AM* (*SD*)	% Above reference level	N	Median (min and max)	*GM* (*SD*)	*AM* (*SD*)	% Above reference level
Metal foodware (ppm)	4%	89	1710 (< *LOD*, 12,100)	1545 (3.4)	2452 (2251)	96%	50	1735 (< *LOD*, 17,400)	1378 (4.6)	2732 (3175)	94%
Paint on large surfaces (µg/cm^2^)	20%	64	4.3 (< *LOD*, 1033)	4.2 (18.4)	71.2 (162.5)	2%	34	32.9 (< *LOD*, 550)	11.1 (23.4)	125.4 (170.2)	0%
Spice (ppm)	27%	84	8 (< *LOD*, 2378)	13 (7.2)	1292 (371.9)	38%	44	436 (< *LOD*, 4139)	175 (11.1)	841 (1021)	84%
Soil (ppm)	0%	59	17 (7, 52)	17 (1.4)	18 (7.4)	0%	17	22 (14, 1990)	31 (3.2)	142 (476.5)	6%

Only one household had a reading of lead in paint on a large surface that exceeded the reference level of 1000 µg/cm^2^. This US standard was originally established in 1992 and may not be most reflective of our current understanding of lead risk exposure. The New York City Department of Housing Preservation and Development adopted a new standard of 500 µg/cm^2^ to be implemented in 2025 (NYC Housing Preservation & Development, 2024). Applying this standard, 4 households (4% of households with paint readings) exceeded the threshold. However, it should be noted that nearly 80% of households with paint readings had at least one reading with some amount of detectable lead. Furthermore, the units of µg/cm^2^ and ppm are not interchangeable, so these results may not be representative of the prevalence of paints above India’s regulatory standard of 90 ppm available for purchase or in use in the state.

Toys were assessed in 61 households; 25% of these households had toys that exceeded the 100 ppm threshold, with a maximum reading of more than 4300 ppm. In 61% of the households, the maximum toy lead levels were below the *LOD*. For this reason, toys were excluded from further statistical analysis.

Nine out of a total of 17 tested ceramic samples exceeded 100 ppm. Similar to metal foodware, there are no recommended limits for total lead in ceramics (only leaching tests), so the 100 ppm threshold is based on current research. However, the small sample size and the fact that ceramic cookware items were not commonly identified in the households potentially limit the representativeness of the results.

Out of 4 cosmetic samples identified, all were below 20 ppm, the Bureau of Indian Standard’s limit for lead in cosmetics. Nevertheless, caution is warranted in interpreting these results due to the very small sample size.

Lead content in drinking water was evaluated from a representative subset of homes at which HBAs were conducted, encompassing various sources such as hand pumps, government supply, and borewells. Out of 25 samples, only one exceeded India’s drinking water standard for lead of 10 ppb with a concentration of 17 ppb, and half of all samples fell below the *LOD* of 5 ppb.

Elevated lead content in metal foodware and spices were common among the sampled houses. The characteristics of the lead levels in these household environmental samples according to the *BLL* of the children (greater than or equal to 10 µg/dL versus below 10 µg/dL) are presented in Table 4.

### Association between environmental sources and elevated BLL

Table 5 presents the association between lead in different environmental samples and the odds of a child having a *BLL* ≥ 10 µg/dL. In the crude analysis, the odds of elevated *BLL* are significantly associated with lead content in spices (crude *OR* = 1.40, 95% *CI* 1.24, 1.61) and in soil (crude *OR* = 4.17, 95% *CI* 1.58, 14.11). After adjusting for the child’s age, sex, and demographic factors, the odds of elevated *BLL* are significantly associated only with lead content in spices (*aOR* = 1.35, 95% *CI* 1.17, 1.58). This means when the lead content in spice doubles, the odds of a child having elevated a *BLL* will be 1.4 times higher. Our finding is similar to findings from an earlier study in Patna, Bihar, by Brown et al. (2022), which also observed significant associations between elevated *BLL*s and high levels of lead in turmeric and soil collected from outside households among children living in communities distant from lead-related industries.

**Table 5:** Associations between environmental sources and elevated *BLL*s (*OR* = odds ratio, *Pb* = lead)

Variable	Unadjusted OR (95% *CI*)	*p*-value	Adjusted *OR* (95% *CI*)	*p*-value
Pb in metal foodware	0.96 (0.80–1.15)	0.63	1.09 (0.88–1.36)	0.44
Pb in paint on large surface	1.08 (0.98–1.19)	0.13	1.03 (0.91–1.16)	0.66
Pb in spice	1.40 (1.24–1.61)	< 0.01	1.35 (1.17–1.58)	< 0.01
Pb in soil	4.17 (1.58–14.11)	0.01	2.21 (1.09–9.91)	0.14

### Lead levels in rural versus urban homes

**Fig. 3:**
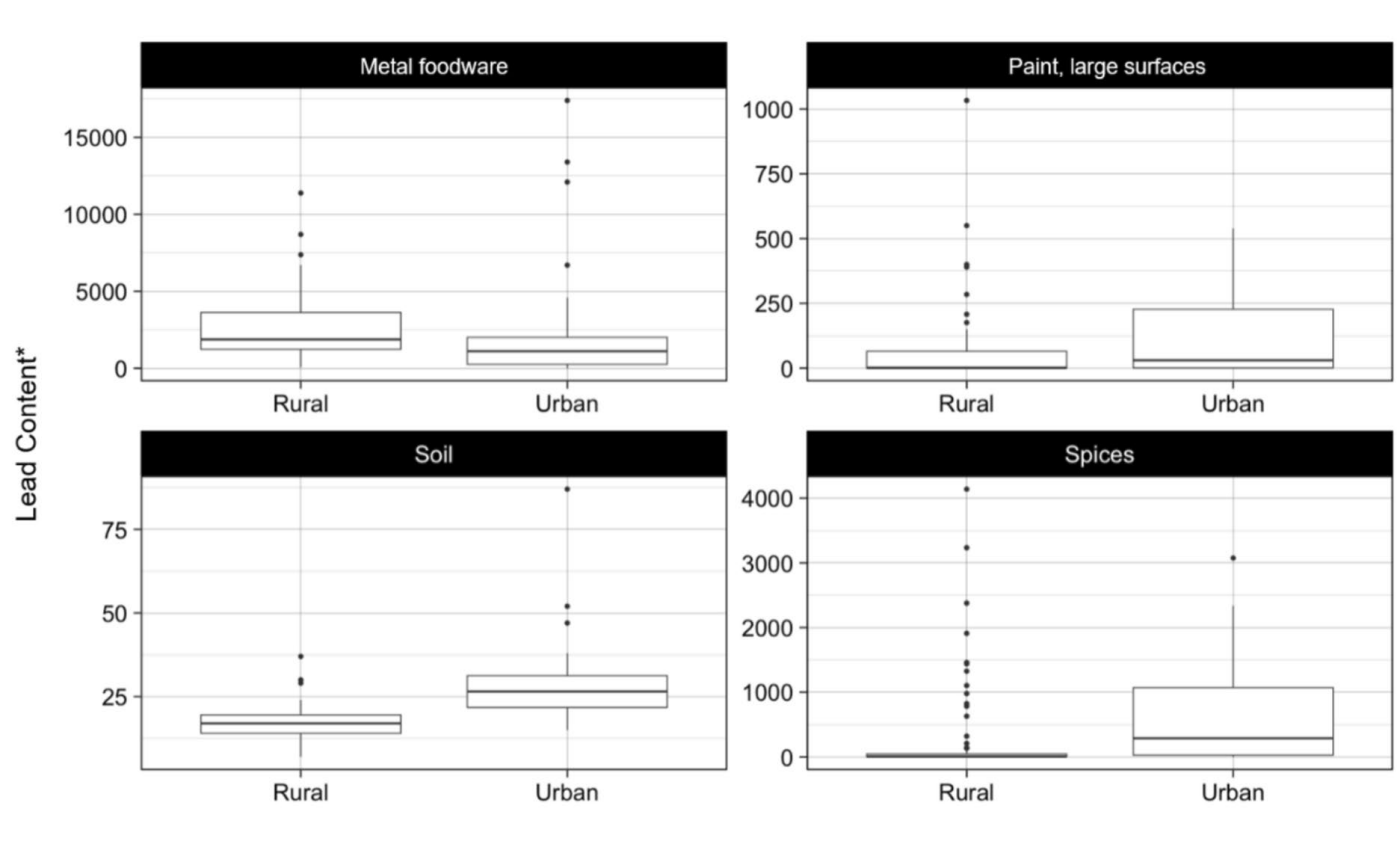
Lead content in environmental samples by urbanicity. * Lead content is expressed in µg/cm^2^ for paint and in ppm for metal foodware, soil, and spices. Note: lead in soil content above 1500 ppm (*N* = 1) not shown

In Table 6, we compare lead levels in environmental samples collected from homes located in urban and rural areas. Applying a Wilcoxon rank sum test shows that lead levels in metal foodware used in rural households were significantly higher than those used in urban households (69% higher median level), potentially reflecting a greater dependence on informally produced cookware. In contrast, urban households reported significantly higher lead levels in paint (21 times higher), spices (36 times higher), and soil (59% higher) than rural households (Fig. 3). These urban disadvantages may reflect greater use and availability of commercial paints, differences in the supply chain for spices, and the historic deposition of lead particles in soil from decades of combustion of leaded gasoline in areas with dense traffic.

**Table 6:** Lead levels in environmental samples by household urbanicity

Environmental sample	Rural	Urban	*p*-value*
	Median (*IQR*)	Median (*IQR*)	
Metal foodware (ppm)	1875 (1240, 3640)	1110 (260, 2020)	< 0.001
Paint on large surface (µg/cm^2^)	1.4 (0.1, 65)	30 (0.8, 227)	0.016
Spice (ppm)	8 (2.1, 47)	288 (27, 1072)	< 0.001
Soil (ppm)	17 (14, 20)	27 (22, 34)	< 0.001

^*^Wilcoxon rank sum test

### Impact

In summary, our assessments identified a high prevalence of elevated lead levels in metal foodware and spices among households tested in the state of Bihar. We found a positive and statistically significant association between lead levels in spices collected in homes and elevated *BLL*s among Bihari children. The concern regarding lead in spices has been previously highlighted in the city of Patna and in other regions in South Asia (Brown et al., 2022; Forsyth et al., 2024). While a small amount of lead can naturally occur in spices due to varying lead content in soil, the levels observed in this study suggest intentional adulteration. Research from Bangladesh indicates that this practice aims to enhance color for greater appeal in the market (Forsyth et al., 2019). The issue of lead in metal foodware emerges as a significant concern from our data, reflecting a growing body of literature from other low- and middle-income countries (Mathee & Street, 2020; Weidenhamer et al., 2017). Lead may be found in cookware that is manufactured from recycled and waste metals (Mathee & Street, 2020; Weidenhamer et al., 2023). Predicting the extent of lead transfer from a specific pot to food is challenging, and there are currently no international standards for total lead content in metal foodware. However, simulated cooking scenarios in laboratory settings have found that pots with comparable lead content can leach quantities of lead exceeding recommended intake values (Fellows et al., 2025; Street et al., 2020; Weidenhamer et al., 2017). While less widespread, elevated lead levels were also identified in specific toys and ceramic items and in paint in one household, which could pose a risk to individual children.

### Strengths and limitations

This study assessed lead levels in a wide range of environmental samples and consumer products in households in Bihar, India and paired this data with biological samples. Households of children with high *BLL*s (above 20 µg/dL) and a random subset of households with low to moderate *BLL*s were evaluated. The random selection of households for the original blood lead testing study from which we drew a subset for this study provided good representation of the state population, not only of communities with known environmental lead contamination.

Limitations should be acknowledged. Although high *BLL*s and lead levels exceeding standards in several sample categories were observed, the overall sample size is fairly small and may have limited the study’s power to detect small or moderate effect sizes. The limited sample size in certain categories, such as cosmetics and ceramics, precludes definitive conclusions. For some environmental samples such as lead in paint on large surfaces, the percentage of samples with levels under detection limits was also high and limited the variation in exposure level among samples. For this analysis, we used the maximum reading for each item category when there were multiple readings for a single household; therefore, any heterogeneity in that category within a single household was not well captured.

Moreover, contribution of lead exposure from the home environment to blood lead levels is likely modified by the child’s behavior (e.g., handwashing, frequency of spice intake, and time spent indoors), home condition (e.g., chipping paint and frequency of mopping the floor), and children’s exposure outside of home (e.g., playing outside or in school or daycare). *BLL*s in children are indicative of relatively recent exposures, while actual exposure to household lead sources may not be temporally correlated.

## Conclusions

The results of home-based lead exposure assessments reveal the prevalence of high lead levels in common household items, particularly cooking spices and metal foodware, across the state of Bihar, India, adding to existing research on exposure sources identified in the capital of Patna and expanding our understanding for the wider region. These findings highlight the strong need for timely interventions to protect residents, especially children, from avoidable lead exposure. In the case of spices, we identified a positive and statistically significant association between increasing lead concentrations and children’s *BLL*s. Therefore, enhanced monitoring and enforcement of existing spice safety rules is critical. The implementation of policies on lead content in metal cookware and an associated monitoring and enforcement approach would represent a proactive step in mitigating this potential source of exposure while research continues to characterize the extent of leaching and the contribution of dietary lead. These actions, combined with health education to raise awareness about lead hazards and promote protective measures, are imperative to mitigate lead exposures and ultimately reduce childhood *BLL*s.

## Data Availability

The datasets used and/or analyzed during the current study are available on specific request only.

## Declarations

All authors have read, understood, and have complied as applicable with the statement on “Ethical responsibilities of Authors” as found in the Instructions for Authors.”

## References

[CR1] Ali Sultan, S. A., Ahmed Khan, F., Wahab, A., Fatima, B., Khalid, H., Bahader, A., Safi, S. Z., Selvaraj, C., Ali, A., Alomar, S. Y., & Imran, M. (2023). Assessing leaching of potentially hazardous elements from cookware during cooking: A serious public health concern. *Toxics,**11*(7), 640. 10.3390/toxics11070640 37505605 10.3390/toxics11070640 PMC10386729

[CR2] Ansari, J. A., Mahdi, A. A., Malik, P. S., & Jafar, T. (2020). Blood lead levels in children living near an informal lead battery recycling workshop in Patna, Bihar. *Journal of Health & Pollution,**10*(25), Article 200308. 10.5696/2156-9614-10.25.200308 32175179 10.5696/2156-9614-10.25.200308 PMC7058140

[CR3] Attina, T. M., & Trasande, L. (2013). Economic costs of childhood lead exposure in low- and middle-income countries. *Environmental Health Perspectives,**121*(9), 1097–1102. 10.1289/ehp.1206424 23797342 10.1289/ehp.1206424 PMC3764081

[CR4] Baig, J. A., Bhatti, S., Kazi, T. G., & Afridi, H. I. (2019). Evaluation of arsenic, cadmium, nickel and lead in common spices in Pakistan. *Biological Trace Element Research,**187*(2), 586–595. 10.1007/s12011-018-1400-4 29882119 10.1007/s12011-018-1400-4

[CR5] Bellinger, D. C. (2004). *Lead. Pediatrics,**113*(4 Suppl), 1016–1022. 15060194

[CR6] Borah, K. K., Bhuyan, B., & Sarma, H. P. (2010). Lead, arsenic, fluoride, and iron contamination of drinking water in the tea garden belt of Darrang district, Assam, India. *Environmental Monitoring and Assessment,**169*(1–4), 347–352. 10.1007/s10661-009-1176-2 19809882 10.1007/s10661-009-1176-2

[CR7] Bose-O’Reilly, S., & Landrigan, P. J. (2022). Chapter 30 - Metal toxicology in low-income and lower-middle-income countries. In G. F. Nordberg & M. Costa (Eds.), *Handbook on the Toxicology of Metals* (5th ed., pp. 705-729). Academic Press. 10.1016/B978-0-12-823292-7.00018-8

[CR8] Brown, M. J., Patel, P., Nash, E., Dikid, T., Blanton, C., Forsyth, J. E., Fontaine, R., Sharma, P., Keith, J., Babu, B., Vaisakh, T. P., Azarudeen, M. J., Riram, B., & Shrivastava, A. (2022). Prevalence of elevated blood lead levels and risk factors among children living in Patna, Bihar, India 2020. *PLOS Global Public Health,**2*(10), Article e0000743. 10.1371/journal.pgph.0000743 36962532 10.1371/journal.pgph.0000743 PMC10021519

[CR9] Central Drugs Standard Control Organization, Ministry of Health and Family Welfare, Directorate General of Health Services, Government of India. (2020). Cosmetics Rules. https://cdsco.gov.in/opencms/opencms/en/Acts-and-rules/Cosmetics-Rules/

[CR10] Ericson, B., Dowling, R., Dey, S., Caravanos, J., Mishra, N., Fisher, S., Ramirez, M., Sharma, P., McCartor, A., Guin, P., Taylor, M. P., & Fuller, R. (2018). A meta-analysis of blood lead levels in India and the attributable burden of disease. *Environment International,**121*(Pt 1), 461–470. 10.1016/j.envint.2018.08.047 30273869 10.1016/j.envint.2018.08.047

[CR11] Fellows, K. M., Samy, S., Rodriguez, Y., & Whittaker, S. G. (2022). Investigating aluminum cookpots as a source of lead exposure in Afghan refugee children resettled in the United States. *Journal of Exposure Science and Environmental EpidemioloGy,**32*(3), 451–460. 10.1038/s41370-022-00431-y 35501355 10.1038/s41370-022-00431-y PMC9119854

[CR12] Fellows, K. M., Samy, S., & Whittaker, S. G. (2025). Evaluating metal cookware as a source of lead exposure. *Journal of Exposure Science and Environmental EpidemioloGy,**35*(3), 342–350. 10.1038/s41370-024-00686-7 38773235 10.1038/s41370-024-00686-7 PMC12069085

[CR13] Food Safety and Standards Authority of India, Ministry of Health and Family Welfare, Government of India. (2011). Food safety and standards (Contaminants, toxins and residues) regulations. https://www.fssai.gov.in/upload/uploadfiles/files/Contaminants_Regulations.pdf

[CR14] Forsyth, J. E., Nurunnahar, S., Islam, S. S., Baker, M., Yeasmin, D., Islam, M. S., Rahman, M., Fendorf, S., Ardoin, N. M., Winch, P. J., & Luby, S. P. (2019). Turmeric means “yellow” in Bengali: Lead chromate pigments added to turmeric threaten public health across Bangladesh. *Environmental Research,**179*, Article 108722. 10.1016/j.envres.2019.108722 31550596 10.1016/j.envres.2019.108722

[CR15] Forsyth, J. E., Baker, M., Nurunnahar, S., Islam, S., Islam, M. S., Islam, T., Plambeck, E., Winch, P. J., Mistree, D., Luby, S. P., & Rahman, M. (2023). Food safety policy enforcement and associated actions reduce lead chromate adulteration in turmeric across Bangladesh. *Environmental Research,**232*, Article 116328. 10.1016/j.envres.2023.116328 37286126 10.1016/j.envres.2023.116328

[CR16] Forsyth, J. E., Mistree, D., Nash, E., Angrish, M., & Luby, S. P. (2024). Evidence of turmeric adulteration with lead chromate across South Asia. *Science of the Total Environment,**949*, Article 175003. 10.1016/j.scitotenv.2024.175003 39053552 10.1016/j.scitotenv.2024.175003

[CR17] Gleason, K., Shine, J. P., Shobnam, N., Rokoff, L. B., Suchanda, H. S., Ibne Hasan, M. O., Mostofa, G., Amarasiriwardena, C., Quamruzzaman, Q., Rahman, M., Kile, M. L., Bellinger, D. C., Christiani, D. C., Wright, R. O., & Mazumdar, M. (2014). Contaminated turmeric is a potential source of lead exposure for children in rural Bangladesh. *Journal of Environmental and Public Health,**2014*, Article 730636. 10.1155/2014/730636 25214856 10.1155/2014/730636 PMC4158309

[CR18] Keosaian, J., Venkatesh, T., D’Amico, S., Gardiner, P., & Saper, R. (2019). Blood lead levels of children using traditional Indian medicine and cosmetics: A feasibility study. *Global Advances in Health and Medicine,**8*, Article 2164956119870988. 10.1177/2164956119870988 31489260 10.1177/2164956119870988 PMC6709437

[CR19] Kumar, D., Awasthi, S., Mahdi, A. A., Singh, S., Pandey, A. K., Agarwal, G. G., Anish, T. S., A R, S., Kar, S., Nair, S., Mathew, J. L., Bhat, M. A., Mahanta, B. N., Singh, K., & Singh, C. M. (2025). Assessment of blood lead level of school children in 10 cities of India: A cross-sectional study. *Indian Journal of Pediatrics,**92*(2), 131–137. 10.1007/s12098-023-04864-7 37919485 10.1007/s12098-023-04864-7

[CR20] Kumar, R., Kumar, S., Kapley, A., & Gupta, A. (2022). Assessment of lead impact on human and India's response. Council of Scientific and Industrial Research (SIR) and NITI Aayog. https://www.pureearth.org/wp-content/uploads/2022/06/Lead-Report-India-CSIR-NITI-Ayog-June-2022.pdf

[CR21] Lu, Y., Chandan, A. K., Mehta, S., Kushwaha, M., Kumar, A., Ali, M., Srivastava, A., Ghosh, A. K., Bose-O’Reilly, S., Nambiar, L., & Kass, D. (2024). Assessment of prevalence of elevated blood lead levels and risk factors among children and pregnant women in Bihar. *India. ENvironmental Research,**259*, Article 119528. 10.1016/j.envres.2024.119528 38960355 10.1016/j.envres.2024.119528

[CR22] Mahdi, A. A., Ansari, J. A., Agarwal, A., Ahmad, M. K., Siddiqui, S. S., Jafar, T., & Venkatesh, T. (2020). Case of lead poisoning associated with herbal health supplements. *Journal of Health & Pollution,**10*(28), Article 201214. 10.5696/2156-9614-10.28.201214 33324511 10.5696/2156-9614-10.28.201214 PMC7731492

[CR23] Mathee, A., & Street, R. (2020). Recycled aluminium cooking pots: A growing public health concern in poorly resourced countries. *BMC Public Health,**20*(1), 1411. 10.1186/s12889-020-09485-9 32938416 10.1186/s12889-020-09485-9 PMC7495850

[CR24] Ministry of Environment, Forest and Climate Change, Government of India. (2016). Regulation of lead contents in household and decorative paints rules. https://moef.gov.in/environment-protection

[CR25] Mohanty, A., Budhwani, N., Ghosh, B., Tarafdar, M., & Chakravarty, S. (2013). Lead content in new decorative paints in India. *Environment, Development and Sustainability,**15*(6), 1653–1661. 10.1007/s10668-013-9455-z

[CR26] Murray, C. J. L., Aravkin, A. Y., Zheng, P., Abbafati, C., Abbas, K. M., Abbasi-Kangevari, M., Abd-Allah, F., Abdelalim, A., Abdollahi, M., Abdollahpour, I., Abegaz, K. H., Abolhassani, H., Aboyans, V., Abreu, L. G., Abrigo, M. R. M., Abualhasan, A., Abu-Raddad, L. J., Abushouk, A. I., Adabi, M., … Lim, S. S. (2020). Global burden of 87 risk factors in 204 countries and territories, 1990–2019: A systematic analysis for the Global Burden of Disease Study 2019. *The Lancet,**396*(10258), 1223–1249. 10.1016/S0140-6736(20)30752-2 10.1016/S0140-6736(20)30752-2 PMC7566194 33069327

[CR27] NITI Aayog. (2023). India national multidimensional poverty index: A progress review. Government of India. https://www.niti.gov.in/sites/default/files/2023-08/India-National-Multidimentional-Poverty-Index-2023.pdf

[CR28] Nordin, N., & Selamat, J. (2013). Heavy metals in spices and herbs from wholesale markets in Malaysia. *Food Additives & Contaminants. Part B, Surveillance,**6*(1), 36–41. 10.1080/19393210.2012.721140 24786623 10.1080/19393210.2012.721140

[CR29] NYC Housing Preservation & Development, 2024. Lead-based paint. https://www.nyc.gov/site/hpd/services-and-information/lead-based-paint.page. Accessed 16 Apr 2025

[CR30] Rashid, A., Bhat, R. A., Qadri, H., Mehmood, M. A., & Shafiq-Ur-Rehman. (2019). Environmental and socioeconomic factors induced blood lead in children: An investigation from Kashmir India. *Environmental Monitoring and Assessment,**191*(2), 76. 30648205 10.1007/s10661-019-7220-y

[CR31] Rees, N., & Fuller, R. (2020). The toxic truth : Children’s exposure to lead pollution undermines a generation of future potential (2nd edn). Issuing bdy: UNICEF, Pure Earth.

[CR32] Senanayake, M. P., Perera, R., Liyanaarachchi, L. A., & Dassanayake, M. P. (2013). Spices as a source of lead exposure: A market-basket survey in Sri Lanka. *Ceylon Medical Journal,**58*(4), 168–169. 10.4038/cmj.v58i4.6308 24385059 10.4038/cmj.v58i4.6308

[CR33] Street, R. A., Mathee, A., Tanda, S., Hauzenberger, C., Naidoo, S., & Goessler, W. (2020). Recycling of scrap metal into artisanal cookware in the informal sector: A public health threat from multi metal exposure in South Africa. *The Science of the Total Environment,**699*, Article 134324. 10.1016/j.scitotenv.2019.134324 33736189 10.1016/j.scitotenv.2019.134324

[CR34] Thermo Fisher Scientific. (2013). Thermo Scientific Niton XL3T GOLDD Series XRF Analyzers for consumer goods: Elemental limits of detection for consumer goods screening. https://tools.thermofisher.com/content/sfs/brochures/Niton-XL3t-GOLDD-Consumer-Goods-LODs-2013Feb05.pdf. Accessed 16 Apr 2025

[CR35] United States Consumer Product Safety Commission. (2023). Consumer product safety improvement Act, 15 U.S.C. § 1278a. https://uscode.house.gov/view.xhtml?req=granuleid:USC-prelim-title15-section1278a&num=0&edition=prelim

[CR36] United States Department of Housing and Urban Development. (2012). Guidelines for the evaluation and control of lead-based paint hazards in housing. Office of healthy homes and lead lazard control. (2nd edn). https://www.hud.gov/sites/documents/second_edition_2012.pdf

[CR37] United States Environmental Protection Agency. (2024). Updated soil lead guidance for CERCLA sites and RCRA corrective action facilities. https://www.epa.gov/superfund/updated-soil-lead-guidance-cercla-sites-and-rcra-corrective-action-facilities. Accessed 16 Apr 2025

[CR38] Vishwanath, P., Devegowda, D., Prashant, A., Nayak, N., D’souza, V., Venkatesh, T., & Scott, C. (2012). Environmental lead levels in a coastal city of India: The lead burden continues. *Indian Journal of Medical Sciences,**66*(11–12), 260–266. 23897520

[CR39] Weidenhamer, J. D., Fitzpatrick, M. P., Biro, A. M., Kobunski, P. A., Hudson, M. R., Corbin, R. W., & Gottesfeld, P. (2017). Metal exposures from aluminum cookware: An unrecognized public health risk in developing countries. *The Science of the Total Environment,**579*, 805–813. 10.1016/j.scitotenv.2016.11.023 27866735 10.1016/j.scitotenv.2016.11.023

[CR40] Weidenhamer, J. D., Chasant, M., & Gottesfeld, P. (2023). Metal exposures from source materials for artisanal aluminum cookware. *International Journal of Environmental Health Research,**33*(4), 374–385. 10.1080/09603123.2022.2030677 35100934 10.1080/09603123.2022.2030677

[CR41] World Health Organization. (2021). *WHO guideline for the clinical management of exposure to lead*. World Health Organization. 34787987

